# Impact of population structure in the estimation of recent historical effective population size by the software GONE

**DOI:** 10.1186/s12711-023-00859-2

**Published:** 2023-12-04

**Authors:** Irene Novo, Pilar Ordás, Natalia Moraga, Enrique Santiago, Humberto Quesada, Armando Caballero

**Affiliations:** 1 Centro de Investigación Mariña, Universidade de Vigo, Facultade de Bioloxía, 36310 Vigo, Spain; 2 Departamento de Biología Funcional, Facultad de Biología, Universidad de Oviedo, 33006 Oviedo, Spain

## Abstract

**Background:**

Effective population size (*N*_*e*_) is a crucial parameter in conservation genetics and animal breeding. A recent method, implemented by the software GONE, has been shown to be rather accurate in estimating recent historical changes in *N*_*e*_ from a single sample of individuals. However, GONE estimations assume that the population being studied has remained isolated for a period of time, that is, without migration or confluence of other populations. If this occurs, the estimates of *N*_*e*_ can be heavily biased. In this paper, we evaluate the impact of migration and admixture on the estimates of historical *N*_*e*_ provided by GONE through a series of computer simulations considering several scenarios: (a) the mixture of two or more ancestral populations; (b) subpopulations that continuously exchange individuals through migration; (c) populations receiving migrants from a large source; and (d) populations with balanced systems of chromosomal inversions, which also generate genetic structure.

**Results:**

Our results indicate that the estimates of historical *N*_*e*_ provided by GONE may be substantially biased when there has been a recent mixture of populations that were previously separated for a long period of time. Similarly, biases may occur when the rate of continued migration between populations is low, or when chromosomal inversions are present at high frequencies. However, some biases due to population structuring can be eliminated by conducting population structure analyses and restricting the estimation to the differentiated groups. In addition, disregarding the genomic regions that are involved in inversions can also remove biases in the estimates of *N*_*e*_.

**Conclusions:**

Different kinds of deviations from isolation and panmixia of the populations can generate biases in the recent historical estimates of *N*_*e*_. Therefore, estimation of past demography could benefit from performing population structure analyses beforehand, by mitigating the impact of these biases on historical *N*_*e*_ estimates.

**Supplementary Information:**

The online version contains supplementary material available at 10.1186/s12711-023-00859-2.

## Background

Effective population size (*N*_*e*_) [1] is a fundamental parameter that plays a key role in quantifying the impact of genetic drift and inbreeding in a population. Its relevance extends across several fields of study, including conservation genetics, evolutionary biology, and animal and plant breeding [2, 3], and it is regarded as a main indicator for biodiversity conservation [4, 5]. There is a plethora of methods to estimate both the contemporary and the historical effective population sizes from genetic markers [6]. Among the estimators of *N*_*e*_ that require a single sample, the method that is based on the linkage disequilibrium (LD) between genetic markers [7–11] stands out and is considered as highly reliable in a wide range of scenarios [12]. The use of unlinked markers allows the estimation of contemporary *N*_*e*_. However, the availability of a vast amount of linked genetic markers provided by current genotyping and sequencing methods also allows for the estimation of *N*_*e*_ in past generations [13, 14]. A recent refinement of the method to estimate historical *N*_*e*_ from linked markers has proven to be highly accurate for inferring significant population changes that have occurred over the past 100 to 200 generations [15]. This method, which is implemented in the software GONE (available at https://github.com/esrud/GONE), has been evaluated with simulations for a range of scenarios [15–19] and applied to multiple sets of empirical data from different species. The possibility of estimating changes in population size in the recent past is particularly useful in livestock species, for which the start of breeding programs may imply substantial changes in *N*_*e*_. To date, this software has been applied to various breeds of pigs [20], cattle [21, 22], sheep [23, 24], horses [25] and chickens [26, 27], and fish species such as turbot, seabream and seabass [16], Baltic herring [28], pikeperch [29], coho salmon [30], catfish [31] and sailfish [32].

The software GONE provides estimates of the effective population size from the variance of progeny number (*N*_*eVk*_, [19]). For a panmictic Wright–Fisher population, *N*_*eVk*_ = *N*, the number of breeding individuals in the population. The estimates assume that the population under study is closed or has remained isolated for a long period of time. However, admixture and population substructure are almost universal in most species. For example, artificial populations used to start breeding programmes are initially composed of a mixture of other populations [33, 34]. In addition, many populations are made up of subpopulations in the form of herds, colonies, or other types of aggregations [35]. This subdivision of the population leads to genetic differentiation among subpopulations and can be counteracted by migration, an important force of change in allele frequencies [36]. Migration not only produces changes in allele frequencies over time, but also in the magnitude of linkage disequilibrium between loci [37–39]. In fact, for structured populations with migration, there may be differences in the estimates of *N*_*e*_ depending on its definition (e.g. *N*_*e*_ based on inbreeding, on allele frequency variance, on linkage disequilibrium, etc.) [40–44].

The impact of immigration and population structure on the estimates of contemporary *N*_*e*_ and historical population size has been previously studied. Regarding contemporary *N*_*e*_, Waples and England [43] investigated the scenario of a population composed of a number of subpopulations that exchange migrants over time. By means of analytical methods and computing simulations, they showed that the estimates of contemporary *N*_*e*_ are generally robust to equilibrium migration among subpopulations. When individuals from a focal subpopulation are sampled, the estimates of local *N*_*e*_ are generally accurate unless the migration rate between subpopulations is high (higher than 5–10%), in which case *N*_*e*_ estimates converge to the global population *N*_*e*_. In addition, when sampling involves individuals from several subpopulations, the estimates may also converge to the global population *N*_*e*_ [43, 44].

Regarding historical population size, migration is known to generate spurious signals of changes in the size of populations which are kept at a stable size [45–52]. Mazet et al. [50] showed that inferential methods of past population demography, such as the pairwise sequentially Markovian coalescent (PSMC) method [53], which provides estimates of historical population size in the long term, may infer inexistent changes in population size if the populations are structured. Moreover, Santiago et al. [15] carried out some simulations to investigate the bias incurred by their method for the estimation of short-term historical population size in structured populations with migration and admixture. They showed that there was little distortion in *N*_*e*_ estimates when there is a metapopulation established through the mixture of two populations. However, this study was limited to a particular scenario in which the mixture had taken place many generations before the estimation time. In addition, Santiago et al. [15] considered a model of two independent subpopulations that exchange migrants at different rates. If individuals were sampled from the two subpopulations and the migration rate between subpopulations was high, there was no bias in the estimation of the total size of the population. However, when the migration rate was low there were substantial biases in the estimates for recent generations.

In the present study, the objective is to further investigate the impact of admixture and migration on the estimates of recent historical *N*_*e*_ obtained from linkage disequilibrium between markers by the software GONE. To do so, we extended the number of scenarios considered by Santiago et al. [15]. For the generation of synthetic populations, we consider populations with a different number of generations evolving separately and different times of population mixture. In the case of the exchange of migrants between subpopulations, we consider cases of two or ten subpopulations, a wide range of migration rates, and a sampling process made either from several subpopulations or from only one of them. In addition, we consider a model in which a population is continuously receiving migrants from a source population of a much larger size.

We also analyse another source of intrinsic population structuring, which arises from chromosomal inversions, a scenario that has not been previously considered. Chromosomal inversions are regions of the DNA sequence, which have a reverse orientation compared to the normal karyotype and are found in a wide variety of species [54, 55]. During meiosis, crossing-over within the inverted region often leads to nonviable gametic products in individuals that are heterozygous for the inversion, and thus are semi-sterile. Therefore, inversions are deleterious and tend to be lost in most species. However, some species have developed mechanisms that prevent the deleterious impact of inversions, and these are frequently observed on different chromosomes. In fact, in some species, inversions provide an evolutionary advantage to the carrier individuals, because their genome contain non-recombining gene clusters that are involved in adaptation [56–58]. For example, in the genus *Drosophila*, many inversions are observed that can exhibit variations in latitudinal and altitudinal gradients, indicating a role in the adaptation and natural selection of the population to the environmental conditions in which they are found [59–61]. In other cases, inversions are known to be involved in speciation, creating barriers between species (such as reproductive isolation) through linked groups that cause sterility [62]. Large-scale inversions are the most common chromosome rearrangements in plants and animals, with their average size ranging from 130 kb to 100 Mb [62]. Because in heterozygous individuals chromosomal inversions inhibit recombination within the inversion region, some segments within this region will exhibit strong linkage disequilibrium and thus clearly distort the estimation of historical *N*_*e*_ based on it.

## Methods

We used the software SLiM v3 [63] to carry out forward-in-time individual-based simulations. Populations were typically composed of *N* = 500 or 1000 individuals and were run for a total period of 10,000 generations under random mating and discrete generations, accounting for mutation, recombination, genetic drift and migration. The simulated genome consisted of a sequence of 250 Mb where mutations appear at a rate between 1 and 5 × 10^–9^ mutations per nucleotide and generation. Only neutral mutations were considered. The recombination between adjacent nucleotides was set at 10^–8^, implying a uniform recombination rate of 1 cM/Mb, which is an approximate average value obtained in multiple species [64]. In the last simulated generation, 100 individuals were sampled to carry out the analysis of historical *N*_*e*_ estimation. The final results are based on 40 replicates of the simulation for each scenario considered.

### Demographic scenarios

**Fig. 1:**
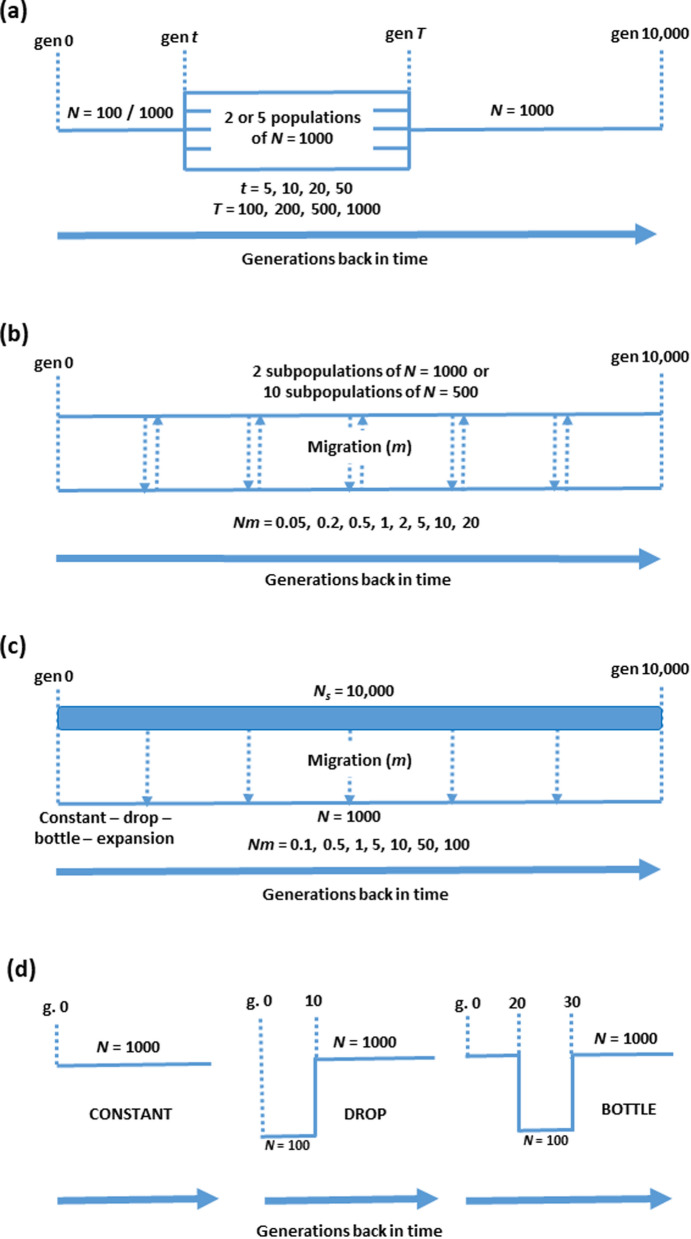
Population scenarios considered. **a** A synthetic population of size *N* individuals was generated *t* generation ago by the mixture of two or five populations of size *N* = 1000 individuals, which diverged *T* generations ago from an ancestral population (*t* < *T*). **b** A population subdivided into two or ten subpopulations was run with continuous migration between them with a reciprocal migration rate *m* per generation. **c** A population with *N* = 1000 individuals was maintained and received migrants from a larger source population of size *N*_*s*_ = 10,000 individuals at a rate *m* per generation. The recipient population was either kept at a constant population size, or suffered from a recent decline in size, a recent population bottleneck and recovery, or an expansion. **d** A closed population was run in which individuals carried a large chromosomal inversion. The population was either kept at a constant size or was subjected to a recent drastic drop, or a bottleneck and later recovering the previous size. Generation 0 is the current one, and generations are shown back in time in all cases

Four scenarios were considered for simulations (Fig. 1): (a) a synthetic population generated by the mixture of different populations; (b) a population subdivided into two or ten subpopulations with continuous migration between them; (c) a population receiving migrants from a large source population; and (d) a closed population in which individuals carry a large chromosomal inversion. For the synthetic population scenario, we simulated a population with a constant census size of *N* = 1000 individuals, which diverged at time *T* = 100, 200, 500 or 1000 generations ago into two or five populations of *N* = 1000 individuals each, and were subsequently (at time *t* = 5, 10, 20 or 50 generations ago) mixed into a single synthetic population of *N* = 100 or 1000 individuals (Fig. 1a). For the migration scenario, a population was subdivided into two subpopulations of *N* = 1000 individuals or ten subpopulations of *N* = 500 individuals, and these were maintained with a reciprocal migration rate per generation (*m*) ranging from 0.0001 to 0.04 (*Nm* from 0.05 to 20) (Fig. 1b). In the last simulated generation, two types of sampling were performed: sampling 100 individuals from a single subpopulation, or sampling an equal number of individuals from each subpopulation to obtain a sample size of 100, i.e., 50 per subpopulation in the scenario of two subpopulations, and 10 per subpopulation in the scenario of 10 subpopulations. For the source-sink scenario (Fig. 1c), a population with *N* = 1000 individuals received migrants at a rate *m* between 0.0001 and 0.1 in each generation (*Nm* ranging from 0.1 to 100) from a larger population with a constant size *N*_*s*_ = 10,000 individuals. The recipient population was either maintained with a constant population size or suffered from drastic changes (a decline in size, a bottleneck and recovery, or a population expansion) in the most recent generations.

In addition, we simulated a closed population with a constant size *N* = 1000 individuals carrying a large inversion of 50 Mb in the middle of the simulated sequence (Fig. 1d). Recombination was precluded within the inverted region in individuals that are heterozygous for the inversion. In order to maintain an intermediate frequency for the inversion, a frequency-dependent selection model was assumed following the model suggested in the SLiM manual (version 3.6): the effect of the inversion on fitness was assumed to be 1 − [(*q* − 0.5) × 0.2], where *q* is the frequency of the inversion. Thus, for *q* = 0.5 the inversion effect on fitness is 1, and this effect is higher than 1 (the inversion is advantageous) if *q* < 0.5, and lower than 1 (the inversion is deleterious) if *q* > 0.5. In this scenario, two demographic changes (other than constant population size) were also considered: a drop in population size to *N* = 100 individuals that occurred at generation 10 before present, and a bottleneck to *N* = 100 individuals that occurred at generation 30 before present to later recover the initial population size. In addition, we considered the case when the frequency of the inversion was low (about 0.15) by introducing the inversion in the population in the last 50 generations, so that the time elapsed was not sufficient for the inversion to reach an intermediate frequency.

### Estimation of the historical *N*_*e*_ and population structure

The program GONE [15] was used to obtain estimates of the historical effective population size from linkage disequilibrium using all available pairs of single nucleotide polymorphisms (SNPs) at distances between *c* = 0.05 and 0.001 Morgan in samples of 100 individuals. The data was assumed to be unphased. First, an in-house C program was used to generate the *map* and *ped* format files from the SLiM output files. Two estimates of the historical *N*_*e*_ were obtained by combining the data of 20 SLiM replicates (mimicking 20 chromosomes), which involved a total number of SNPs of 0.5 to 1 million for the analysis. The two obtained estimates were very close to each other and were averaged.

The estimation of *N*_*e*_ was also carried out after discarding the inverted genomic region using the programme PLINK v.1.9 [65], which was also used to perform an analysis of principal components in the populations. The software STRUCTURE [66] was used to visualise the population structure generated in the different scenarios. The *ped* files were converted to STRUCTURE input files using the programme PGDSpider [67].

As explained above, GONE provides estimates of *N*_*e*_ from the variance of progeny number (*N*_*eVk*_) without accounting for migration. Therefore, the simulation results presented in the next section are compared to this expected *N*_*eVk*_, which, for the assumed simulation model, equals *N*, the number of breeding individuals. When several contemporary populations or subpopulations are simulated, the total expected *N*_*eVk*_ is considered to be the sum of the *N*_*eVk*_ of all the populations. Thus, the *N*_*eVk*_ of a subpopulation or the sum of several subpopulations are the references with which all estimations of historical *N*_*e*_ are compared.

## Results

### Synthetic populations

**Fig. 2:**
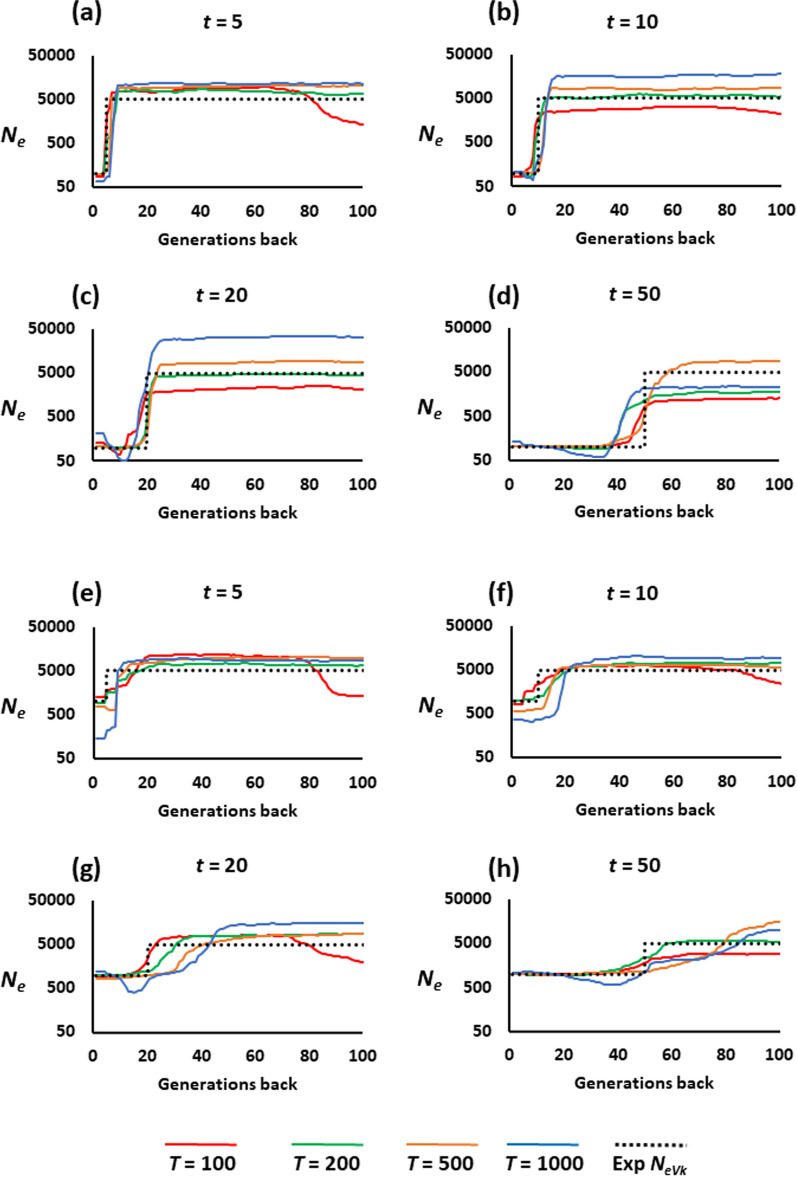
Estimates of the historical effective population size (*N*_*e*_) obtained by the software GONE for a synthetic population. The synthetic population was generated *t* generations ago (**a** and **e** *t* = 5, **b** and **f** *t* = 10, **c** and **g** *t* = 20, **d** and **h** *t* = 50) from the mixture of five populations which diverged *T* generations ago from an ancestral population. The expected *N*_*e*_ from the variance of the progeny number (Exp *N*_*eVk*_) is shown as a black dotted line. Before mixing, the expected *N*_*eVk*_ is the sum for all populations (5000). After mixing, the expected *N*_*eVk*_ = 100 for **a**–**d**, and 1000 for **e**–**h**. The sample size at generation zero is 100 individuals

Figure 2 shows the estimation of the historical *N*_*e*_ when a synthetic population has been created *t* generations ago from the mixture of five populations which have diverged independently *T* generations ago. If the synthetic population has a small size (*N* = 100 individuals; Fig. 2a–d), the estimation of the time of admixture is rather accurate, with more error incurred when the time of admixture took place a long time ago (*t* = 20 or 50 generations). The estimated current *N*_*e*_ is generally close to the true *N*_*eVk*_, whereas the ancestral total size of the five populations combined together (*N*_*eVk*_ = 5000) is usually overestimated, although it can also be underestimated, particularly if the time of admixture is long (*t* = 50 generations; Fig. 2d).

When the synthetic population has a large size (*N* = 1000 individuals; Fig. 2e–h the estimated *N*_*e*_ is more variable, with the time of admixture generally overestimated. If the ancestral populations have been isolated for a long time (*T* = 1000 generations; blue lines), the most recent *N*_*e*_ is greatly biased downwards when the time of admixture is short (*t* = 5 or 10 generations). For longer times since admixture (*t* = 20 or 50), there is a reduction in the estimated *N*_*e*_, which progressively reaches the true recent *N*_*eVk*_ in the most recent generations.

Additional file 1: Figure S1 shows the results for synthetic populations generated from the mixture of two ancestral populations, instead of five, and provides analogous conclusions to those of Fig. 2.

### Migration between subpopulations

**Fig. 3:**
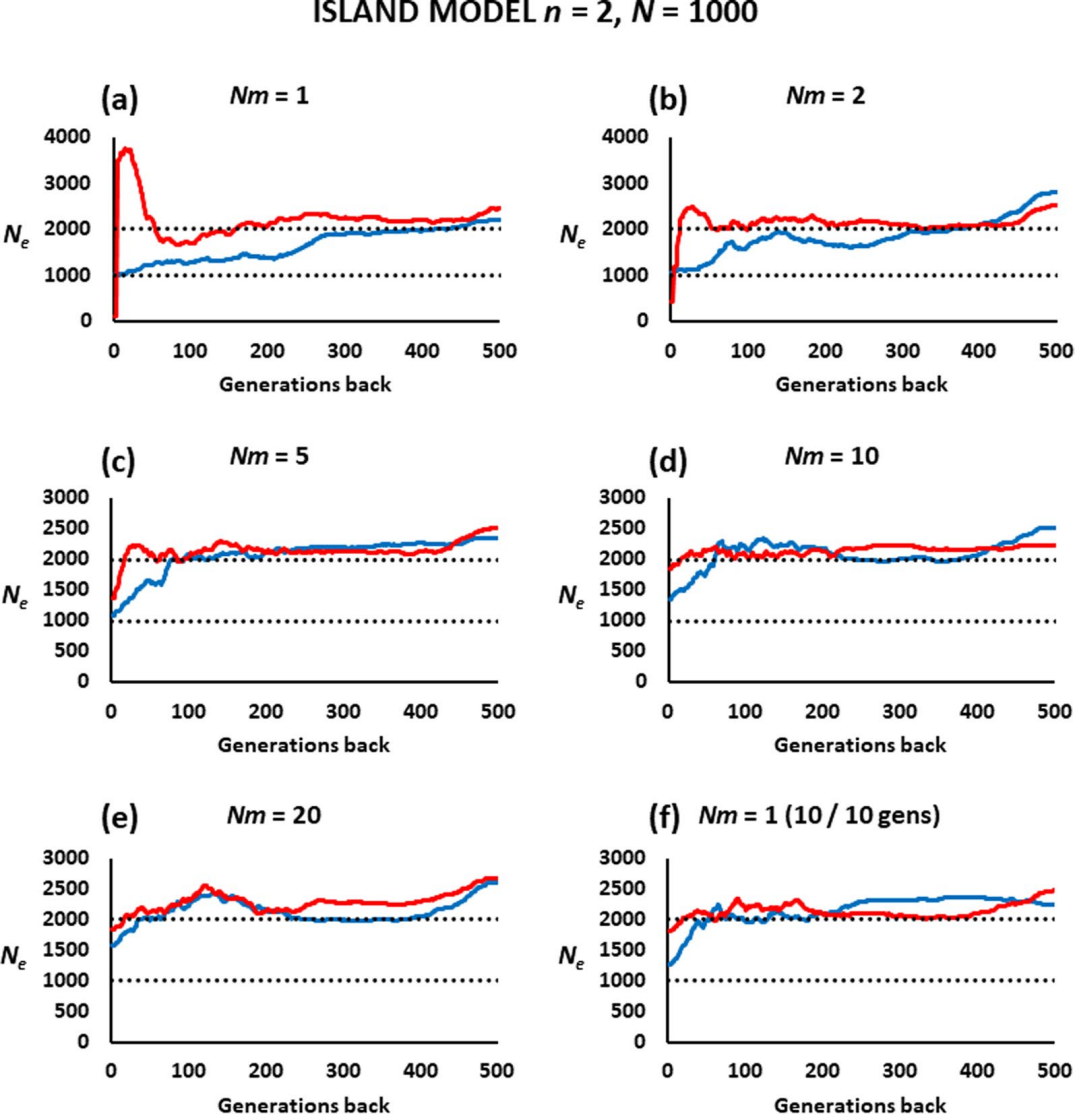
Estimates of the historical effective population size (*N*_*e*_) obtained by the software GONE for a population subdivided into two subpopulations. The *n* = 2 subpopulations of size *N* = 1000 individuals each have continuous reciprocal migration at a rate *m* per generation (**a** *Nm* = 1 migrant each generation, **b** *Nm* = 2 migrants each generation, **c** *Nm* = 5 migrants each generation, **d** *Nm* = 10 migrants each generation, **e** *Nm* = 20 migrants each generation, **f** *Nm* = 1 = 10 migrants each 10 generations). The two black dotted lines show the expected *N*_*eVk*_ of one subpopulation (lower line) or both together (upper line). The red lines correspond to estimates obtained from 100 individuals sampled at generation zero from the two subpopulations (half of each), whereas the blue lines correspond to estimates obtained from 100 individuals sampled from a single subpopulation

Figure 3 shows the estimated *N*_*e*_ values in a population subdivided into two subpopulations, between which there is a continuous flow of individuals at different rates. The analysis can be made using sampled individuals from a single subpopulation (blue lines), or from both subpopulations (red lines). In the first case, the estimated contemporary *N*_*e*_ is close to the true *N*_*eVk*_ if the rate of migration is low (*Nm* ≤ 5), and tends to increase back in time as the migration rate between the two subpopulations is higher. For high migration rates (*Nm* ≥ 10), the contemporary *N*_*e*_ of a subpopulation gets closer to the sum of the *N*_*eVk*_ of the two subpopulations, as expected.

If the sampled individuals belong to both subpopulations (red lines in Fig. 3), the estimated *N*_*e*_ is close to the *N*_*eVk*_ of the whole population when migration rates are high (*Nm* ≥ 5). However, a substantial bias is observed when migration rates are low, with a very large increase in *N*_*e*_ over time and a sudden drop in the most recent generations. A scenario with *m* = 0.01 every ten generations (Fig. 3f), which would imply an average *m* = 0.001 per generation, gives results closer to those of the scenario with *m* = 0.01 per generation (*Nm* = 10; Fig. 3d) than to those with *m* = 0.001 per generation (*Nm* = 1; Fig. 3a).

The scenario of subpopulations with a very low migration rate between them generates substantial structuring in the population, as illustrated in Additional file 1: Fig. S2. This allows for a possible solution to the above biased estimation. Additional file 1: Fig. S3 shows an analysis of principal components for the scenarios in Fig. 3. If the structure of the population is clear, one could possibly select individuals from only one of the groups (as marked with a circle in the graphs of Additional file 1: Fig. S3) and use these for *N*_*e*_ estimation. This would be equivalent to sampling within a single subpopulation. The corresponding resulting estimates are also shown in Additional file 1: Fig. S3, indicating that the large artefacts observed in Fig. 3 are removed and the estimates are reasonably accurate for values of *Nm* ≤ 10 and the 50 most recent generations.

**Fig. 4:**
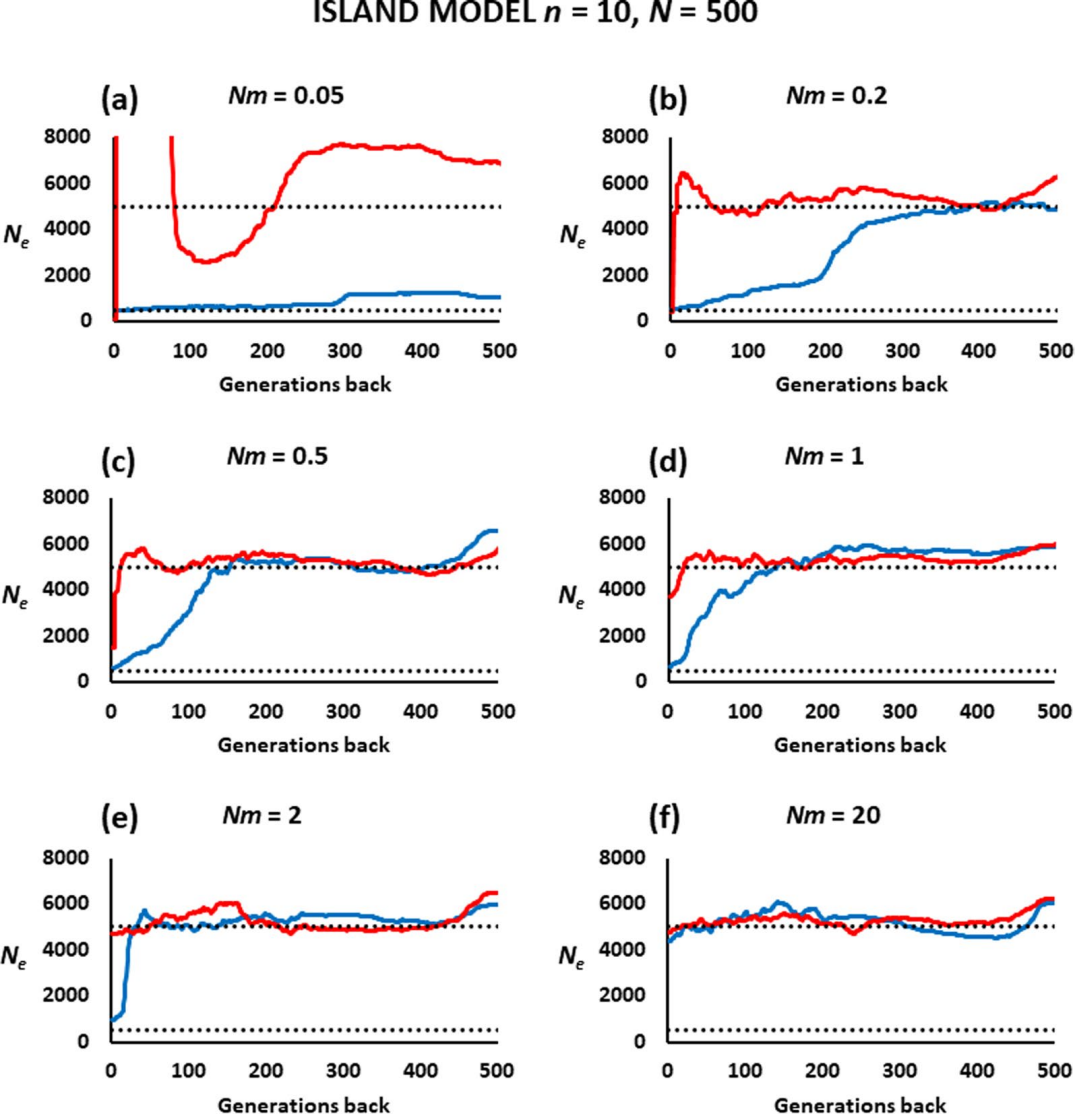
Estimates of the historical effective population size (*N*_*e*_) obtained by the software GONE for a population subdivided into ten subpopulations. The *n* = 10 subpopulations of size *N* = 500 individuals each, have continuous reciprocal migration between each other (island model) at a migration rate *m* per generation (**a** Nm = 0.05 = 1 migrant each 20 generations, *b* Nm = 0.2 = 1 migrant each 5 generations, **c** Nm = 0.5 = 1 migrant each 2 generations, **d** Nm = 1 migrant each generation, e Nm = 2 migrants each generation, f Nm = 20 migrants each generation). The two black dotted lines show the expected *N*_*eVk*_ of one subpopulation (lower line) or all of them together (upper line). The red lines correspond to estimates obtained from 100 individuals sampled at generation zero from the ten subpopulations (10 individuals from each subpopulation), whereas the blue lines correspond to estimates obtained from 100 individuals sampled from a single subpopulation

Figure 4 shows the scenario of an island model with *n* = 10 subpopulations of *N* = 500 individuals and a range of migration rates among them. The results are qualitatively similar to those of Fig. 3, with some differences in time scale. When samples are taken from one subpopulation (blue lines), the estimated *N*_*e*_ becomes closer to the subpopulation’s *N*_*eVk*_ in fewer generations than for the two-subpopulation model for a given migration rate (e.g., cf. Figs. 3a and 4d for *Nm* = 1). When samples are taken from all subpopulations (red lines), the ancestral total *N*_*eVk*_ = 5000 is well estimated across time for all scenarios except for very low migration rates (*Nm* = 0.05–0.2) and in the very recent generations, for which there is a drastic drop in the estimated *N*_*e*_.

### Source-sink scenario

**Fig. 5:**
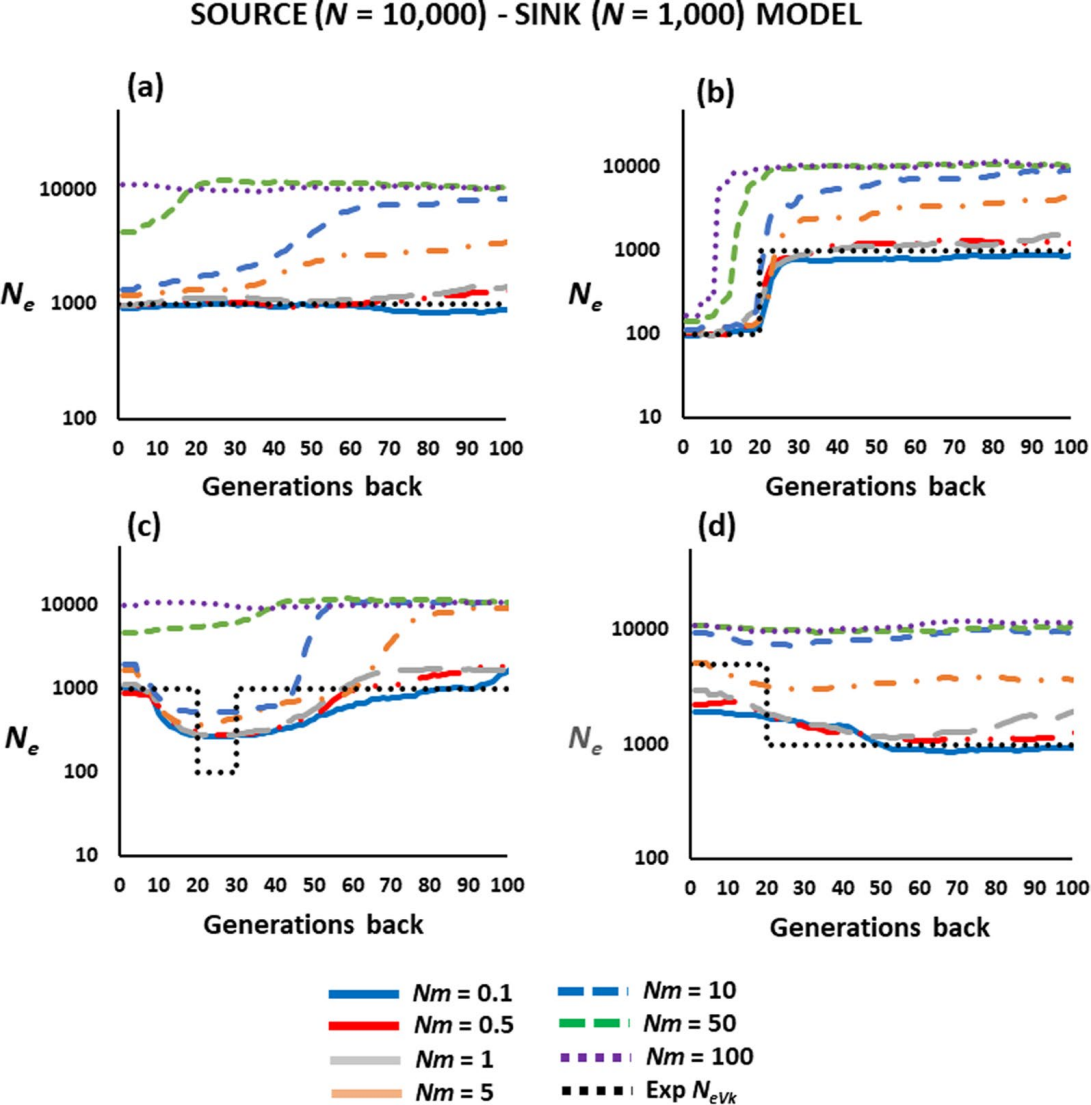
Estimates of the historical effective population size (*N*_*e*_) obtained by the software GONE for a population with *N* = 1000 individuals which continuously receives migrants from a larger source population (*N*_*s*_ = 10,000) at a rate *m* per generation. The recipient population was either **a** kept at a constant population size, **b** subjected to a recent decline in size, **c** subjected to a recent population bottleneck and recovery, or **d** subjected to a recent expansion. The black dotted line shows the expected *N*_*e*_ from the variance of the progeny number (Exp *N*_*eVk*_) of the recipient population

Results for the source-sink scenario are shown in Fig. 5. When the migration rate into the target population is low (*Nm* = 0.1–1), the estimation of the historical *N*_*e*_ is generally not affected by migration, except in the recent expansion scenario (Fig. 5d), in which the expansion is underestimated. However, when the migration rate from the large source population is higher (*Nm* ≥ 5), there is an increasing impact on the estimates as the migration rate increases. If the recipient population has a constant size (Fig. 5a), the impression is that *N*_*e*_ typically declines over time. If a drastic drop has occurred, its estimated time is more recent than the true time for high migration rates (*Nm* ≥ 50; Fig. 5b). Finally, a recent bottleneck is still detected even with *Nm* as large as 10 (Fig. 5c), but a recent expansion would be undetected for high migration rates (Fig. 5d). With a very high migration rate (*Nm* = 100), the estimated *N*_*e*_ is close to the *N*_*eVk*_ of the source population (10,000) with only a decline in *N*_*e*_ in the last ten generations in the drop scenario (Fig. 5b).

### Chromosomal inversions

**Fig. 6:**
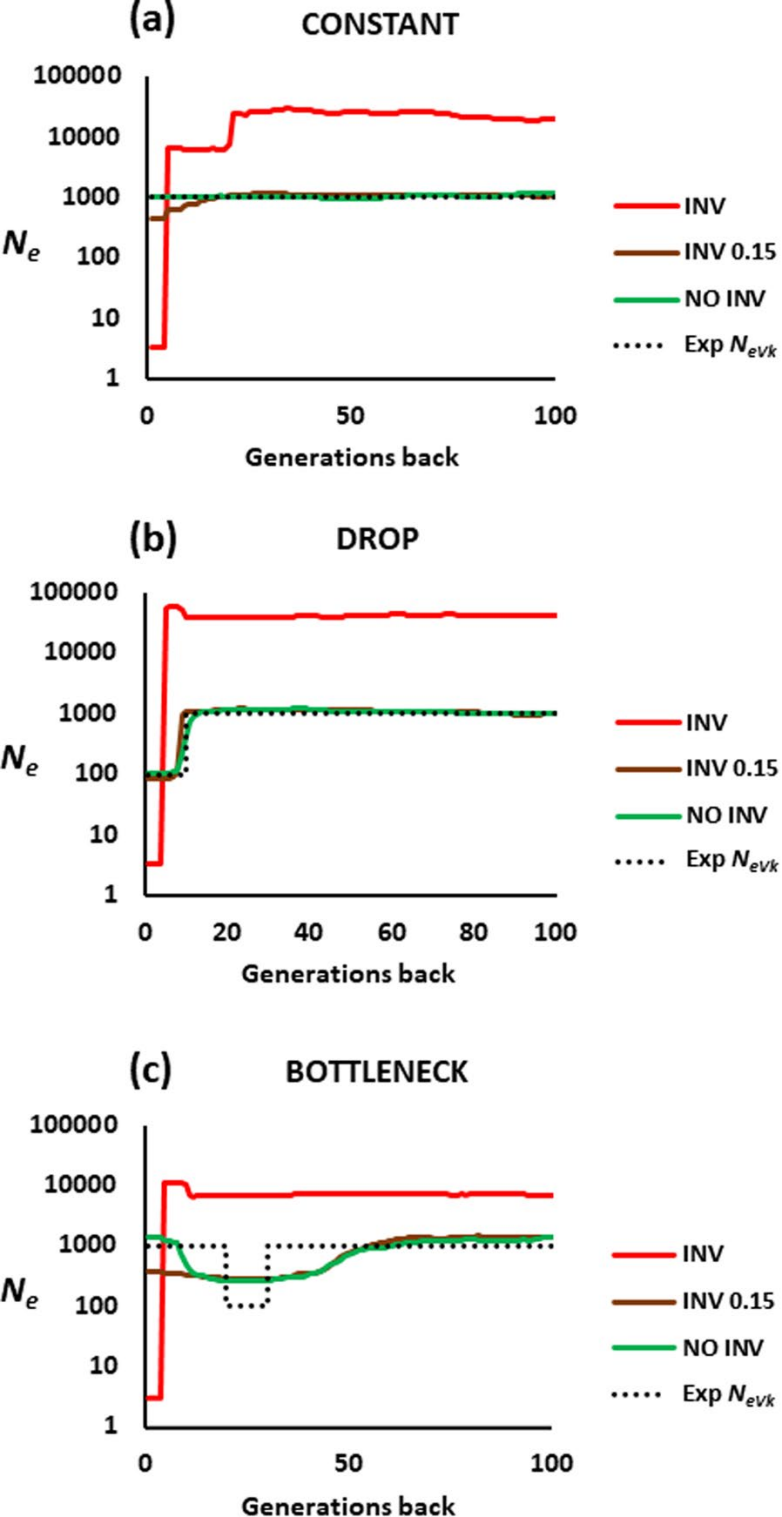
Estimates of the historical effective population size (*N*_*e*_) obtained by the software GONE for a closed population with *N* = 1000 individuals carrying a large chromosomal inversion. The population was either **a** kept at a constant size, **b** subjected to a recent drastic drop, or **c** subjected to a bottleneck and later recovering of the previous size. The red lines correspond to the scenario where the inversion is present in the population at an intermediate frequency (around 0.5). The brown lines show the case for which the inversion is at a low frequency (0.15) in the population. The green lines refer to the estimated *N*_*e*_ values when the inverted region is discarded in the analysis. Finally, the black dotted line shows the expected *N*_*e*_ from the variance of the progeny number (*N*_*eVk*_)

Finally, Fig. 6 presents the estimated historical *N*_*e*_ values in a population in which a large genomic inversion is segregating. Figure 6a shows the results for a constant population size. When the frequency of the inversion is intermediate (red line), the estimation of the historical *N*_*e*_ is highly biased, with an outcome very similar to that of Figs. 3 and 4 with a low migration rate. This occurs because the inversion also generates a population structure, as shown in Additional file 1: Fig. S4. This artefact is also seen in the scenarios with a drop in *N* (Fig. 6b) or a population bottleneck (Fig. 6c). When the frequency of the inversion is lower (around 0.15), the impact on the recent estimated *N*_*e*_ is much smaller (brown lines). Fortunately, when the inverted genomic region can be identified and the SNPs within this region are discarded in the analysis, the estimation becomes quite accurate (green lines).

## Discussion

Migration and population structuring have been shown to generate biases in the estimates of contemporary effective population size, obtained from the temporal change in allele frequencies [68], from linkage disequilibrium between markers [43], or from continuously distributed populations [69]. These biases have also been shown to occur in the historical estimates of population size [46–52]. In this study, we have investigated the impact of population structuring on the estimation of historical effective population size from linkage disequilibrium between linked markers using GONE, a novel tool capable of accurately estimating the historical effective population size in the recent past [15]. GONE is expected to provide estimates of *N*_*e*_ from the variance of progeny number (*N*_*eVk*_) assuming the population is closed, so that it does not account for population structuring. This is a problem common to all methods used to infer past population size from a single contemporary sample of individuals. We considered alternative models which can be encountered in different situations and potentially affect the estimates obtained. The first scenario examined the model that involved the mixture of populations, either naturally or artificially, to generate a synthetic population. This is a common procedure in the creation of base populations to start breeding programmes in aquaculture settings [33, 34]. Our results suggest that the estimation of the historical *N*_*e*_, either before or after the creation of the synthetic population, is relatively accurate, but biases of the estimates increase when the synthetic population has a large size and the time of divergence between the mixed populations is very long. The linkage disequilibrium generated when populations are mixed [37] explains why the estimates deviate further from the simulated value when the divergence is more ancient, since the longer the populations have remained separated, the greater the differentiation between allele frequencies at the time of convergence. It also explains why estimates of *N*_*e*_ that deviate further from the true *N*_*eVk*_ were obtained when the separation resulted in a larger number of populations (5 versus 2), as it leads to the fixation of allele variants in five different populations. Santiago et al. [15] considered the case in which two populations of size *N* = 1000 separated by 100 generations confluence in a single one 50 generations in the past, showing a good estimation of *N*_*eVk*_, which is in agreement with our result shown by the red line in Additional file 1: Fig. S1h for *t* = 50 and *T* = 100. However, when the mixed populations have been separated by a longer period of time (*T* = 500 or 1000 generations), the estimates are not so precise. In this case, recent estimates are particularly biased downwards when the synthetic population has been created a short time ago (*t* = 5 or 10 generations) (Fig. 2e, f and Additional file 1: Fig. S1e, f). This occurs because the strong initial linkage disequilibrium that occurred after mixing is decreased by recombination [43], so that a number of generations are needed to recover from the effects of mixing. This can be clearly seen in the results of Fig. 2g, h and (see Additional file 1: Fig. S1g, h) corresponding to *t* = 20 or 50 and *T* = 500 or 1000 (orange and blue lines), for which *N*_*e*_ declines considerably around the time of mixing to further reach an estimate of contemporary *N*_*e*_ very close to the true *N*_*eVk*_. In general, the estimates of recent *N*_*e*_ obtained by GONE are more precise than ancient estimates, as the footprint of linkage slowly disappears over time [15, 16].

In the above results, it is assumed that the current population is known (or deduced) to be the result of a mixture from two or more ancestral populations in the past, for example in the case of the creation of a synthetic population to start a breeding program. However, if the existence of a previous mixture was not known, the results from the estimation of the historical *N*_*e*_ could be interpreted as those of a closed population which has suffered from a drop in size in the past. This should be acknowledged in the interpretation of empirical results.

The second scenario investigated considered a population structured in two or ten subpopulations with continuous reciprocal migration between them. The sampled individuals can be taken only from a single (focal) subpopulation. In this case, the estimates of local recent *N*_*e*_ are generally close to the true *N*_*eVk*_ when the migration rates are low, but tend towards the *N*_*e*_ of the entire population as migration rates increase (Figs. 3 and 4). This is consistent with the results of previous studies showing that linkage disequilibrium estimates of local contemporary *N*_*e*_ are accurate for migrations rates *m* lower than about 0.05 to 0.1, but increase substantially for higher migration rates [43, 44]. Regarding ancient estimates of historical *N*_*e*_, the effect is greater than for the recent *N*_*e*_ (Figs. 3 and 4), giving the impression of a continuous linear decline of *N*_*e*_ over time while, in fact, this is kept invariable over time.

The estimates of *N*_*e*_ can also be obtained from a sample belonging to the different subpopulations (Figs. 3 and 4), for example when the boundaries of the subpopulations are unclear or when sampling is made at locations and times where the individuals of different subpopulations are intermixed [43]. The impact of sampling on the estimation of *N*_*e*_ in structured populations has been reported by different studies (e.g. [50]). In this situation, the estimates of the historical *N*_*e*_ show a very large bias with substantial overestimation, leading to a sudden drop in the most recent generations. This is clear for migration rates up to *m* = 0.005, which corresponds to *Nm* = 5 in the simulations of Fig. 3 and up to *m* = 0.002 (*Nm* = 1) in the simulations of Fig. 4, and disappears for higher migration rates, for which the estimated *N*_*e*_ is close to the *N*_*eVk*_ value of the entire population. Santiago et al. [15] showed that this undesired effect can be improved by considering pairs of SNPs in the analysis with recombination rates lower than the maximum value of 0.5. A value of 0.05 was recommended and considered by default in the software GONE, and this is what we used in all the analyses of the present paper. Even so, the biases incurred are evident for low values of gene flow between subpopulations. This can make it difficult to distinguish whether there has been a real sharp decline in *N*_*e*_ in the most recent generations in a closed population or whether it is an artefact caused by population admixture. This type of result can be seen in some recent analyses such as those in two or three Croatian sheep breeds analysed by Drzaic et al. [23] and one of the horse breeds analysed by Criscione et al. [25]. In addition, a similar result has been found in experimental populations of *Drosophila melanogaster* maintained in bottles with 50% mixture between them every generation [19]. Thus, these results should be interpreted with caution. One possible solution, as previously suggested [10, 43], is to use population structure or assignment methods to try to identify particular migrants or a clear structuring of the population. In extreme cases, such as those illustrated in Additional file 1: Fig. S3, where the individuals of the two subpopulations can be identified, independent analyses for each of the groups can provide estimates of historical *N*_*e*_ without such an artefact.

The third scenario considered a focal population receiving migrants from a source population with a considerably larger size (Fig. 5). As expected, for low migration rates (*Nm* < 1) the estimates of *N*_*e*_ reflect the *N*_*eVk*_ of the local population, whereas for very high migration rates (*Nm* = 100) the estimated *N*_*e*_ reflects that of the source population. However, for intermediate values of migration (1 < *Nm* < 50), the ancient estimated *N*_*e*_ progressively declines from values closer to that of the source population to values closer to that of the recipient population if this latter has a constant size (Fig. 5a). Again, some of the results suggest a decline in historical *N*_*e*_ over time. This decline could be interpreted as the local population getting shrunk in size or some events of genetic drift causing this reduction. However, this is not the case, and it is just the reflection of continuous migration from a larger source population. When the recipient population suffers from a real decline in size, a bottleneck, or a recent expansion, this change can be observed although its timing or magnitude may not be correct (Fig. 5b–d).

Our results are consistent with previous studies that addressed the impact of migration on the estimation of past demography [46–52]. Mazet et al. [50] introduced a parameter, the “inverse instantaneous coalescence rate” (IICR), which reflects the true population size in closed panmictic populations, but can be strongly disconnected from the real demographic history in admixed populations. They tested their method with simulation data and analyses using the pairwise sequentially Markovian coalescent (PSMC) method [53], which provides estimates of historical population size in the long term and actually infers the IICR curves. Their analyses showed that, in a population subdivided in several subpopulations with migration between each other (island model), the demographic inference made with PSMC and predicted by IICR, is a decline in population size with time although the size of all the subpopulations is kept constant over time. Thus, the inference of a population decline with empirical data could be due to a true demographic change but also to population structuring with migration [50–52].

Our results in Fig. 4c, considering an island model of 10 subpopulations with *N =* 500 diploid individuals (i.e. whole population size of 5000 individuals), and a migration rate *m* = 0.001 (*Nm* = 0.5), is analogous to that considered by Mazet et al. [50]. Although the results from both studies are qualitatively similar, there are several large quantitative differences between them. In the estimations obtained by PSMC or the IICR prediction, when the sampling of individuals is made from a single subpopulation (Figure 4a in Mazet et al. [50]) the estimated population size from PSMC and IICR overpredicts the whole population size of 5000 individuals (estimates around 8000), and drops since the last 3000 generations onwards, down to the subpopulation size (500 individuals). The estimates by GONE for the same model (blue line in Fig. 4c) show a more accurate prediction of the ancestral global population of 5000 individuals over the last 500 generations, dropping down to the subpopulation size (500 individuals) only for the last 100 generations. When samples were taken from different subpopulations (Figure 4b in Mazet et al. [50]), the ancestral inferred population size was again an overestimation, further increasing in the last 1000 generations towards much larger values of tens of thousands of individuals. In contrast, the estimates by GONE, shown by the red line in Fig. 4c predicted well the ancestral global population of 5000 individuals, and the drastic decline toward values closer to the subpopulation size occurred only in the most recent 10 generations.

The above results suggest that GONE provides more accurate short-term estimates of historical population size than PSMC and IICR in scenarios that involve migration between subpopulations. The software PSMC provides estimates of past population size from one sampled diploid individual, which can be extended to a few more individuals by MSMC [70]. Santiago et al. [15] showed that, for relatively recent timespans of about 200 generations back in time, MSMC [70] and Relate [71] are generally unable to detect changes in population size. These mutation-recombination-based methods to infer past population size are expected to be more accurate for long-term evolutionary estimations than for very recent times. In contrast, GONE, which provides estimates for up to about 500 generations at most, is only reliable for periods of about 100 to 200 generations.

The final scenario that we considered refers to the presence of chromosomal inversions in the population. By inhibiting recombination in the inverted region in heterozygotes [72], inversions alter the linkage disequilibrium relationships between loci, creating genetic structuring by separating genomes into two groups (with and without the inversion) with no genetic exchange within the inverted region (see Additional file 1: Fig. S4). This leads to a bias in the estimation of *N*_*eVk*_ (Fig. 6). We simulated a rather extreme scenario, where the inverted region represents a large part of the genome sequence analysed and the inversion is maintained at an intermediate frequency by frequency-dependent selection. In that situation, a substantial bias in the estimates of historical *N*_*e*_ is observed. Because of the lack of recombination in the inverted region, distant loci maintain a strong linkage disequilibrium, and the software interprets this as a drastic reduction in the recent *N*_*e*_. This type of generated bias is very close to that observed above for a subdivided population with a very low migration rate between subpopulations and sampling from both of them (Figs. 3a, b and 4a, b). Fortunately, the bias is much smaller when the inversion is at low frequency, and can be removed altogether when the inverted region can be identified and discarded from the analysis (Fig. 6). Furthermore, there may be some genetic exchange in the central regions of the inversion [59, 73], so that, for old inversions, the linkage disequilibrium between loci within the inversion may not be as extreme as simulated here.

## Conclusions

In summary, the software GONE is a novel tool that can accurately estimate the historical effective population size from the variance of progeny number of populations as long as the assumptions made for the model (a panmictic closed population) are achieved. The alteration of the linkage disequilibrium pattern caused by population structure and chromosomal inversions may prevent the correct estimation of historical *N*_*eVk*_. A pattern that is repeatedly observed because of population structuring is a reduction in *N*_*e*_ over time, sometimes as a very drastic drop in the most recent generations, and sometimes as a continuous linear decline. Thus, empirical results showing these patterns should be interpreted with caution. Although some actions can be taken to mitigate these biases, such as performing an analysis of population structure beforehand and the removal of genomic regions involved in chromosomal inversions, further theoretical developments are needed to account for population structure.

### Supplementary Information

**Additional file 1: Figure S1.** Estimates of the historical effective population size (*N*_*e*_) obtained by the software GONE for a synthetic population. The synthetic population was generated *t* generations ago (**a** and **e** *t* = 5, **b** and **f** *t* = 10, **c** and **g** *t* = 20, **d** and **h** *t* = 50) from the mixture of two populations which diverged *T* generations ago from an ancestral population. The expected *N*_*e*_ from the variance of the progeny number (Exp *N*_*eVk*_) is shown as a black dotted line. Before mixing, the expected *N*_*eVk*_ is the sum for all populations (2000). After mixing, the expected *N*_*eVk*_ = 100 for **a**–**d**, and 1000 for **e**–**h**. The sample size at generation zero is 100 individuals. **Figure S2.** Analysis of population structuring obtained with the software STRUCTURE for a replicate of the scenarios shown in Fig. 3. The scenario corresponds to a population subdivided into two subpopulations of size *N* = 1000 each with continuous migration between them and a reciprocal migration rate *m* per generation. The 100 individuals analysed are sampled from the two subpopulations (half of each), and refer to the cases with migration rates per generation *Nm* = 1 (a), 2 (b), 5 (c), 10 (d), 20 (e), and 1 (*m* = 0.01 every 10 generations) (f). **Figure S3.** Principal component analysis of the analysed individuals corresponding to a simulated replicate of the scenario in Fig. 3, regarding two subpopulations run with continuous reciprocal migration between them at a rate *m* per generation. Graphs (a–d): representation of the two main principal components for each of the scenarios. The circles show the groups of individuals recognised as belonging to each of the two subpopulations. Graphs (e–g): estimates of the historical effective population size (*N*_*e*_) obtained by the software GONE considering only the group of individuals ascribed to a single subpopulation (a–c). The black dotted line shows the expected *N*_*e*_ from the variance of progeny number (*N*_*eVk*_) of the subpopulation. **Figure S4.** Analysis of population structuring obtained with the software STRUCTURE for a replicate of the scenario of a closed population. The closed population had *N* = 1000 individuals carrying a large chromosomal inversion at an intermediate frequency (corresponding to the red line of panel (a) in Fig. 6). The graph shows the 100 sampled individuals that are carriers of the inversion or non-carriers (green or red), as well as the heterozygous individuals for the inversion (bars with both colours).

## Data Availability

Computer scripts used in the study are available at Github address https://github.com/irene-novo-g/Structure-Ne.

## References

[1] Wright S (1931). Evolution in mendelian populations. Genetics.

[2] Frankham R, Ballou JD, Briscoe DA (2010). Introduction to conservation genetics.

[3] Caballero A (2020). Quantitative genetics.

[4] Hoban S, Bruford M, Jackson JDU, Lopes-Fernandes M, Heuertz M, Hohenlohe PA (2020). Genetic diversity targets and indicators in the CBD post-2020 global biodiversity framework must be improved. Biol Conserv.

[5] Frankham R (2021). Suggested improvements to proposed genetic indicator for CBD. Conserv Genet.

[6] Nadachowska-Brzyska K, Konczal M, Babik W (2022). Navigating the temporal continuum of effective population size. Methods Ecol Evol.

[7] Hill WG (1981). Estimation of effective population size from data on linkage disequilibrium. Genet Res (Camb)..

[8] Waples RS (2006). A bias correction for estimates of effective population size based on linkage disequilibrium at unlinked gene loci. Conserv Genet.

[9] Waples RS, Do CHI (2010). Linkage disequilibrium estimates of contemporary ne using highly variable genetic markers: a largely untapped resource for applied conservation and evolution. Evol Appl.

[10] Luikart G, Ryman N, Tallmon DA, Schwartz MK, Allendorf FW (2010). Estimation of census and effective population sizes: the increasing usefulness of DNA-based approaches. Conserv Genet.

[11] Wang J, Santiago E, Caballero A (2016). Prediction and estimation of effective population size. Heredity (Edinb)..

[12] Gilbert KJ, Whitlock MC (2015). Evaluating methods for estimating local effective population size with and without migration. Evolution.

[13] Hayes BJ, Visscher PM, McPartlan HC, Goddard ME (2003). Novel multilocus measure of linkage disequilibrium to estimate past effective population size. Genome Res.

[14] Tenesa A, Navarro P, Hayes BJ, Duffy DL, Clarke GM, Goddard ME (2007). Recent human effective population size estimated from linkage disequilibrium. Genome Res.

[15] Santiago E, Novo I, Pardiñas AF, Saura M, Wang J, Caballero A (2020). Recent demographic history inferred by high-resolution analysis of linkage disequilibrium. Mol Biol Evol.

[16] Saura M, Caballero A, Santiago E, Fernández A, Morales-González E, Fernández J (2021). Estimates of recent and historical effective population size in turbot, seabream, seabass and carp selective breeding programmes. Genet Sel Evol.

[17] Novo I, Santiago E, Caballero A (2022). The estimates of effective population size based on linkage disequilibrium are virtually unaffected by natural selection. PLoS Genet.

[18] Reid BN, Pinsky ML (2022). Simulation-based evaluation of methods, data types, and temporal sampling schemes for detecting recent population declines. Integr Comp Biol.

[19] Novo I, Pérez-Pereira N, Santiago E, Quesada H, Caballero A (2023). An empirical test of the estimation of historical effective population size using *Drosophila melanogaster*. Mol Ecol Resour.

[20] Krupa E, Moravčíková N, Krupová Z, Žáková E (2022). Assessment of the genetic diversity of a local pig breed using pedigree and SNP data. Genes (Basel)..

[21] Jin H, Zhao S, Jia Y, Xu L (2022). Estimation of linkage disequilibrium, effective population size, and genetic parameters of phenotypic traits in Dabieshan cattle. Genes (Basel)..

[22] Magnier J, Druet T, Naves M, Ouvrard M, Raoul S, Janelle J (2022). The genetic history of Mayotte and Madagascar cattle breeds mirrors the complex pattern of human exchanges in Western Indian Ocean. G3 (Bethesda).

[23] Drzaic I, Curik I, Lukic B, Shihabi M, Li MH, Kantanen J (2022). High-density genomic characterization of native Croatian sheep breeds. Front Genet.

[24] Djokic M, Drzaic I, Shihabi M, Markovic B, Cubric-Curik V (2023). Genomic diversity analyses of some indigenous Montenegrin sheep populations. Diversity (Basel)..

[25] Criscione A, Mastrangelo S, D’Alessandro E, Tumino S, Di Gerlando R, Zumbo A (2022). Genome-wide survey on three local horse populations with a focus on runs of homozygosity pattern. J Anim Breed Genet.

[26] Gao C, Du W, Tian K, Wang K, Wang C, Sun G (2023). Analysis of conservation priorities and runs of homozygosity patterns for Chinese indigenous chicken breeds. Animals (Basel)..

[27] Liu C, Wang D, He Y, Liang W, Li W, Wang K (2023). Population structure and genetic diversity analysis of “Yufen 1” H line chickens using whole-genome resequencing. Life.

[28] Atmore LM, Martínez-García L, Makowiecki D, André C, Lõugas L, Barrett JH (2022). Population dynamics of Baltic herring since the viking age revealed by ancient DNA and genomics. Proc Natl Acad Sci USA.

[29] De Los Ríos-Pérez L, Druet T, Goldammer T, Wittenburg D (2022). Analysis of autozygosity using whole-genome sequence data of full-sib families in pikeperch (*Sander lucioperca*). Front Genet.

[30] Martinez V, Dettleff PJ, Galarce N, Bravo C, Dorner J, Iwamoto RN (2022). Estimates of effective population size in commercial and hatchery strains of coho salmon (* Oncorhynchus kisutch* (Walbaum, 1792)). Animals (Basel)..

[31] Coimbra MRM, Farias RS, da Silva BCNR, Blanco A, Hermida M, Caballero A (2023). A genetic linkage map of the threatened catfish *Lophiosilurus alexandri*: inferences on effective population size. Aquac Fish.

[32] Lopes da Silva Ferrette B, Coimbra RTF, Winter S, De Jong MJ, Williams SM, Coelho R (2023). Seascape genomics and phylogeography of the sailfish (*Istiophorus platypterus*). Genome Biol Evol.

[33] Holtsmark M, Sonesson AK, Gjerde B, Klemetsdal G (2006). Number of contributing subpopulations and mating design in the base population when establishing a selective breeding program for fish. Aquaculture.

[34] Fernández J, Toro MA, Sonesson A, Villanueva B (2014). Optimizing the creation of base populations for aquaculture breeding programs using phenotypic and genomic data and its consequences on genetic progress. Front Genet.

[35] Hartl DL, Clark AG (1997). Principles of population genetics.

[36] Wang J, Whitlock MC (2003). Estimating effective population size and migration rates from genetic samples over space and time. Genetics.

[37] Nei M, Li WH (1973). Linkage disequilibrium in subdivided populations. Genetics.

[38] Sinnock P (1975). The Wahlund effect for the two-locus model. Am Nat.

[39] Pfaff CL, Parra EJ, Bonilla C, Hiester K, McKeigue PM, Kamboh MI (2001). Population structure in admixed populations: effect of admixture dynamics on the pattern of linkage disequilibrium. Am J Hum Genet.

[40] Wang J (1997). Effective size and F-statistics of subdivided populations. II. Dioecious species. Genetics.

[41] Cervantes I, Goyache F, Molina A, Valera M, Gutiérrez JP (2011). Estimation of effective population size from the rate of coancestry in pedigreed populations. J Anim Breed Genet.

[42] Park L (2011). Effective population size of current human population. Genet Res.

[43] Waples RS, England PR (2011). Estimating contemporary effective population size on the basis of linkage disequilibrium in the face of migration. Genetics.

[44] Ryman N, Laikre L, Hössjer O (2019). Do estimates of contemporary effective population size tell us what we want to know?. Mol Ecol.

[45] Wakeley J (2001). The coalescent in an island model of population sub-division with variation among demes. Theor Popul Biol.

[46] Chikhi L, Sousa VC, Luisi P, Goossens B, Beaumont MA (2010). The confounding effects of population structure, genetic diversity and the sampling scheme on the detection and quantification of population size changes. Genetics.

[47] Heller H, Chikhi L, Siegismund HR (2013). The confounding effect of population structure on bayesian skyline plot inferences of demographic history. PLoS One..

[48] Paz-Vinas I, Quéméré E, Chikhi L, Loot G, Blanchet S (2013). The demographic history of populations experiencing asymmetric gene flow: combining simulated and empirical data. Mol Ecol.

[49] Peter BM, Wegmann D, Excoffier L (2010). Distinguishing between population bottleneck and population subdivision by a bayesian model choice procedure. Mol Ecol.

[50] Mazet O, Rodríguez W, Grusea S, Boitard S, Chikhi L (2016). On the importance of being structured: instantaneous coalescence rates and human evolution—lessons for ancestral population size inference?. Heredity (Edinb)..

[51] Rodríguez W, Mazet O, Grusea S, Arredondo A, Corujo JM, Boitard S (2018). The IICR and the non-stationary structured coalescent: towards demographic inference with arbitrary changes in population structure. Heredity (Edinb)..

[52] Chikhi L, Rodríguez W, Grusea S, Santos P, Boitard S, Mazet O (2018). The IICR (inverse instantaneous coalescence rate) as a summary of genomic diversity: insights into demographic inference and model choice. Heredity (Edinb)..

[53] Li H, Durbin R (2011). Inference of human population history from individual whole genome sequences. Nature.

[54] Faria R, Johannesson K, Butlin RK, Westram AM (2019). Evolving inversions. Trends Ecol Evol.

[55] Mérot C, Oomen RA, Tigano A, Wellenreuther M (2020). A roadmap for understanding the evolutionary significance of structural genomic variation. Trends Ecol Evol.

[56] Twyford AD, Friedman J (2015). Adaptive divergence in the monkey flower *Mimulus guttatus* is maintained by a chromosomal inversion. Evolution.

[57] Koch EL, Morales HE, Larsson J, Westram AM, Faria R, Lemmon AR (2021). Genetic variation for adaptive traits is associated with polymorphic inversions in *Littorina saxatilis*. Evol Lett.

[58] Campoy E, Puig M, Yakymenko I, Lerga-Jaso J, Cáceres M (2022). Genomic architecture and functional effects of potential human inversion supergenes. Philos Trans R Soc Lond B Biol Sci.

[59] Hoffmann AA, Sgrò CM, Weeks AR (2004). Chromosomal inversion polymorphisms and adaptation. Trends Ecol Evol.

[60] Kirkpatrick M (2010). How and why chromosome inversions evolve. PLoS Biol.

[61] Dutheil JY (2020). Statistical population genomics.

[62] Wellenreuther M, Bernatchez L (2018). Eco-evolutionary genomics of chromosomal inversions. Trends Ecol Evol.

[63] Haller BC, Messer PW (2019). SLiM 3: forward genetic simulations beyond the Wright–Fisher model. Mol Biol Evol.

[64] Dumont BL, Payseur BA (2008). Evolution of the genomic rate of recombination in mammals. Evolution.

[65] Purcell S, Neale B, Todd-Brown K, Thomas L, Ferreira MA, Bender D (2007). PLINK: a tool set for whole-genome association and population-based linkage analyses. Am J Hum Genet.

[66] Hubisz MJ, Falush D, Stephens M, Pritchard JK (2009). Inferring weak population structure with the assistance of sample group information. Mol Ecol Resour.

[67] Lischer HE, Excoffier L (2012). PGDSpider: an automated data conversion tool for connecting population genetics and genomics programs. Bioinformatics.

[68] Ryman N, Allendorf FW, Jorde PE, Laikre L, Hössjer O (2014). Samples from subdivided populations yield biased estimates of effective size that overestimate the rate of loss of genetic variation. Mol Ecol Resour.

[69] Neel MC, McKelvey K, Ryman N, Lloyd MW, Short Bull R, Allendorf FW (2013). Estimation of effective population size in continuously distributed populations: there goes the neighborhood. Heredity (Edinb)..

[70] Schiffels S, Durbin R (2014). Inferring human population size and separation history from multiple genome sequences. Nat Genet.

[71] Speidel L, Forest M, Shi S, Myers SR (2019). A method for genome-wide genealogy estimation for thousands of samples. Nat Genet.

[72] Pegueroles C, Ordóñez V, Mestres F, Pascual M (2010). Recombination and selection in the maintenance of the adaptive value of inversions. J Evol Biol.

[73] Stevison LS, Hoehn KB, Noor MA (2011). Effects of inversions on within-and between-species recombination and divergence. Genome Biol Evol.

