# Supplementary material for: Impact of population structure in the estimation of recent historical effective population size by the software GONE
Source: Genet Sel Evol. 2023 Dec 4;55:86. doi: 10.1186/s12711-023-00859-2 (PMC10694967; doi:10.1186/s12711-023-00859-2)
Supplement: Supplementary file 1 — Additional file 1: Figure S1. Estimates of the historical effective population size (Ne) obtained by the software GONE for a synthetic population. The synthetic population was generated t generations ago (a and e t = 5, b and f t = 10, c and g t = 20, d and h t = 50) from the mixture of two populations which diverged T generations ago from an ancestral population. The expected Ne from the variance of the progeny number (Exp NeVk) is shown as a black dotted line. Before mixing, the expected NeVk is the sum for all populations (2000). After mixing, the expected NeVk = 100 for a–d, and 1000 for e–h. The sample size at generation zero is 100 individuals. Figure S2. Analysis of population structuring obtained with the software STRUCTURE for a replicate of the scenarios shown in Fig. 3. The scenario corresponds to a population subdivided into two subpopulations of size N = 1000 each with continuous migration between them and a reciprocal migration rate m per generation. The 100 individuals analysed are sampled from the two subpopulations (half of each), and refer to the cases with migration rates per generation Nm = 1 (a), 2 (b), 5 (c), 10 (d), 20 (e), and 1 (m = 0.01 every 10 generations) (f). Figure S3. Principal component analysis of the analysed individuals corresponding to a simulated replicate of the scenario in Fig. 3, regarding two subpopulations run with continuous reciprocal migration between them at a rate m per generation. Graphs (a–d): representation of the two main principal components for each of the scenarios. The circles show the groups of individuals recognised as belonging to each of the two subpopulations. Graphs (e–g): estimates of the historical effective population size (Ne) obtained by the software GONE considering only the group of individuals ascribed to a single subpopulation (a–c). The black dotted line shows the expected Ne from the variance of progeny number (NeVk) of the subpopulation. Figure S4. Analysis of population structuring obtained w [file 12711_2023_859_MOESM1_ESM.docx]

**Impact of population structure in the estimation of recent historical effective population size by the software GONE – Supplementary figures**

**
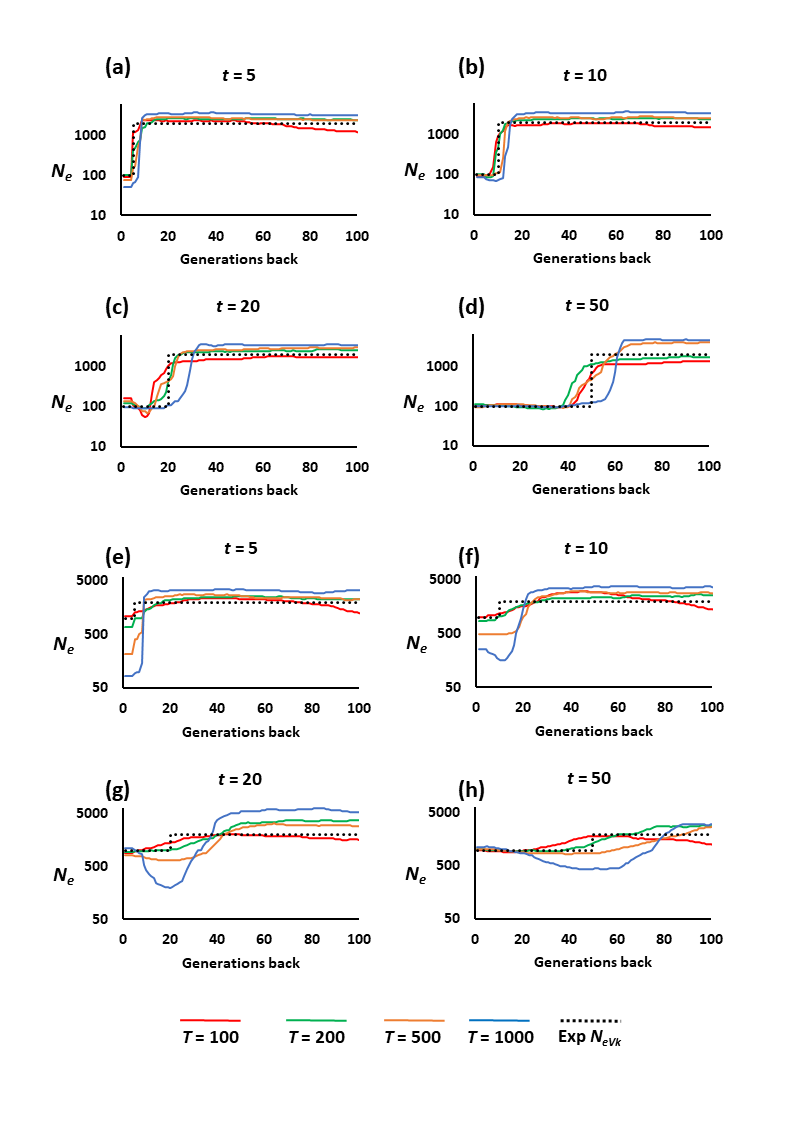
**

**Figure S1.** **Estimates of the historical effective population size (*N_e_*) obtained by the software GONE for a synthetic population.** The synthetic population was generated *t* generation ago from the mixture of two populations which diverged *T* generations ago from an ancestral population. The expected *N_e_* from the variance of progeny number (Exp *N_eVk_*) is shown as a black dotted line. Before mixing, the expected *N_eVk_* is the sum for all populations. The sample size at generation zero is 100 individuals.

**
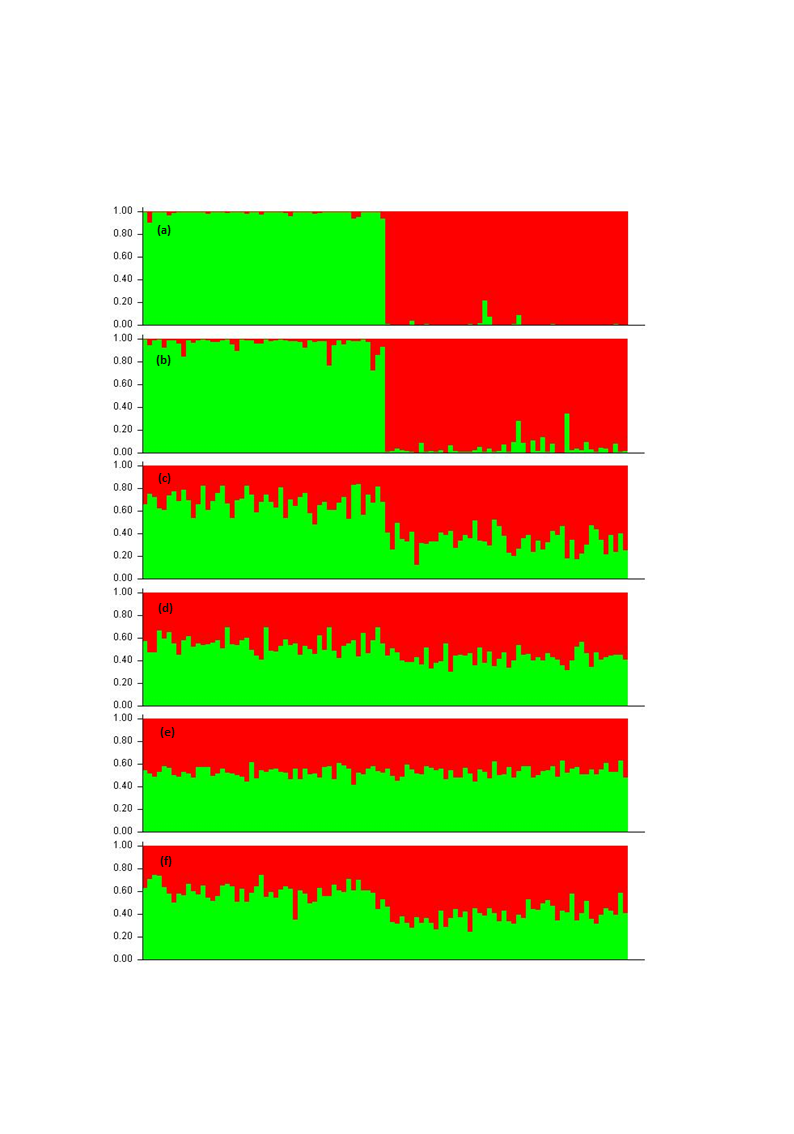
**

**Figure S2.** **Analysis of population structuring obtained with the software STRUCTURE for a replicate of the scenarios shown in Figure 3 of the main text**. The scenario corresponds to a population subdivided into two subpopulations of size *N* = 1000 each with continuous migration between them and a reciprocal migration rate *m* per generation. The 100 individuals analysed are sampled from the two subpopulations (half of each), and refer to the cases with migration rates per generation *Nm* = 1 (a), 2 (b), 5 (c), 10 (d), 20 (e), and 1 (*m* = 0.01 every 10 generations) (f).

**
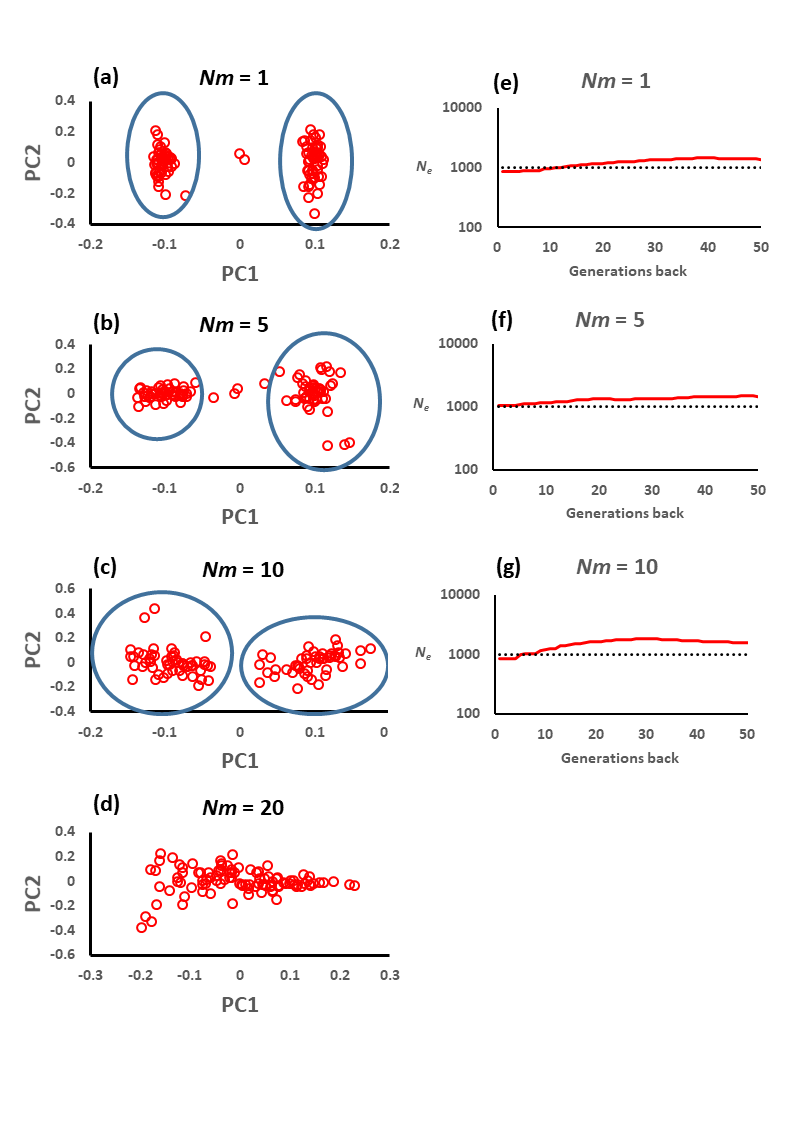
 Figure S3. Principal component analysis of the analysed individuals corresponding to a simulated replicate of the Figure 3 scenario, regarding two subpopulations run with continuous reciprocal migration between them at a rate *m* per generation.** Graphs (a-d): representation of the two main principal components for each of the scenarios. The circles show the groups of individuals recognised as belonging to each of the two subpopulations. Graphs (e-g): estimates of the historical effective population size (*N_e_*) obtained by the software GONE considering only the group of individuals ascribed to a single subpopulation (a-c). The black dotted line shows the expected *N_e_* from the variance of progeny number (*N_eVk_*) of the subpopulation.

**
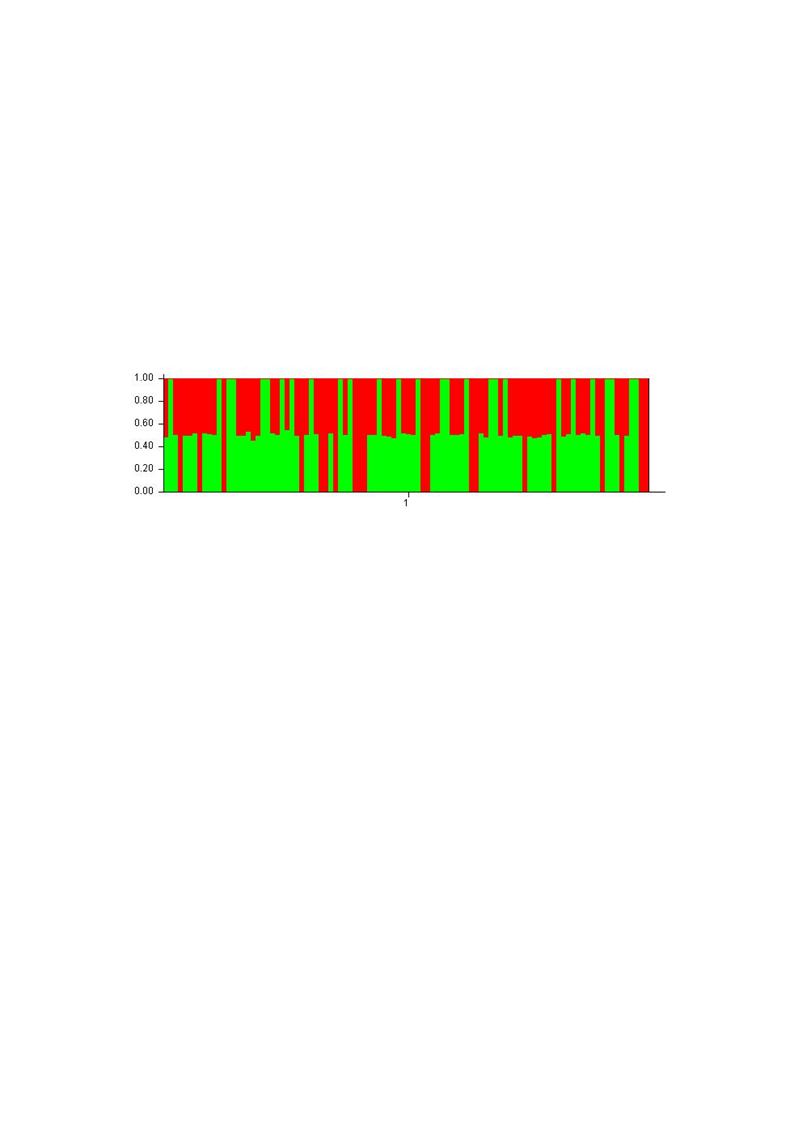
**

**Figure S4. Analysis of population structuring obtained with the software STRUCTURE for a replicate of the scenario of a closed population.** The closed population had *N* = 1000 individuals carrying a large chromosomal inversion at intermediate frequency (corresponding to the red line of panel a in Figure 6 of the main text). The graph shows the 100 sampled individuals carriers of the inversion or non-carriers (green or red), as well as the heterozygous individuals for the inversion (bars with both colours).
